# School Trust and Sense of Belonging: Restoring Bonds and Promoting Well-Being in Schools

**DOI:** 10.3390/ijerph22040498

**Published:** 2025-03-26

**Authors:** Elisabetta Fenizia, Santa Parrello

**Affiliations:** Department of Humanities, University of Naples ‘Federico II’, 80138 Naples, Italy; elisabetta.fenizia@unina.it

**Keywords:** school dropout, school trust, school climate, self-efficacy, adolescence

## Abstract

School dropout is a global issue that compromises individual and societal well-being. Researchers in psychology emphasize that dropout often results from a prolonged erosion of bonds between individuals, schools, and society, especially in socioeconomically disadvantaged contexts. School trust, described as the “connective tissue” within the school system, fosters psychological well-being and is associated with self-esteem, self-efficacy, life satisfaction, and reduced depression. This study aimed to explore the interaction of various relational constructs related to school life, which could be used to improve student well-being and reduce the risk of dropout. A total of 645 high school students from impoverished and high-crime neighborhoods in Naples were involved in the cross-sectional study, investigating the role that school trust plays in relation to positive teaching, self-efficacy, and the sense of belonging. The results indicate that positive teaching significantly enhances the sense of school belonging through the mediating role of students’ trust in teachers. These findings highlight the crucial role of trust as a mediator in strengthening student–school relationships. Schools should prioritize fostering trust by promoting teacher transparency, consistency, and care. Such efforts can enhance students’ sense of belonging, ultimately mitigating dropout risk and restoring their connection with education. This systemic approach is especially vital in contexts with significant socioeconomic challenges.

## 1. Introduction

School dropout can be described as the culmination of a long process of disengagement and detachment from school [1] and as the final result of a progressive deterioration of the bond between young people, schools, and society [2,3]. While it is true that some physical and mental illnesses, particularly chronic ones, contribute to school dropout [4,5], several studies have shown that the opposite can also occur: students who drop out from school are more likely to develop mental, physical, and health disorders [6,7], and this is particularly true for women [8]. High levels of education lead to more dignified and better-paid employment, provide sociopsychological resources (such as self-efficacy and social support), facilitate access to healthcare, and promote healthier lifestyles, thus enhancing health both directly and indirectly [9,10].

In some countries that have recently improved compulsory education laws, the cohorts of young people who benefited have shown indicators of improved health [11]. Estimates even suggest that investments in improving education levels may save more lives than advancements in medicine. For this reason, some authors propose reframing school dropout as a public health issue [12,13] and suggest developing policies aimed not only at individual students, but also at the entire school system. Buland and Mathiesen [14], in particular, highlight the need to implement systemic school policies in order to counter the atomization of interventions enacted by individual schools, as this amplifies initial inequalities: a student who had the misfortune of being born in a marginal context should not have the additional misfortune of being enrolled in a school that does not offer adequate educational resources [14]. This is not to say that there are universal strategies to counter school dropout, which is a multifactorial phenomenon; however, central ministries for education should guarantee that all schools implement early programs capable of preventing psychological dropout from taking root, as it precedes a full-fledged school exit.

In Europe, this phenomenon is measured through the Early School Leaving (ESL) indicator. In particular, students between 18 and 24 years old who drop out of school before completing lower secondary education without enrolling in another training program are defined as early school leavers. In Italy, the ESL percentage stands at 10.5% [15], equivalent to 431,000 students who have left school prematurely. This figure places Italy significantly above the European average of 9.8%. The situation becomes even more concerning when considering that the ESL indicator focuses only on actual dropouts, neglecting issues such as grade retention, academic failure, and educational loss [16].

Many students express the intention of dropping out of school in adolescence, during the period of transition from middle to high school, especially in the Italian school system [17]. This usually occurs after they have manifested different forms of school uneasiness, defined as “an emotional state that might be expressed by a variety of behaviors such as low participation in school activities, low attention, school rejection, disruptive behaviors, problematic relationships with peers and teachers and low academic achievement” [18]. In particular, among the students’ psychological variables correlated with school dropout, the levels of anxiety, depression, stress, and general mental health have been examined [19]. Research on risk factors for school dropout thus intersects research on mental health issues among adolescent students: a study conducted in Italy in 2021 by Donato et al. [20], which involved a broad sample of 15- and 16-year-olds, showed that 1 student out of 5 manifests symptoms of mental health issues, with a more than double proportion for females compared to males (28.7% displaying symptoms of depression among females compared to 10.4% among males). These data compel deep reflection on the characteristics of the school context that might protect or worsen students’ mental health and push them towards dropout: compared to students’ individual and family variables, school variables—which can be defined as the push factors—would be better suited for change through ad hoc interventions [14].

The theoretical model proposed by Tinto [21,22] frames school dropout as a process influenced by events and conditions within the learning environment that shape an individual’s intentions to leave school. Specifically, this model emphasizes the interaction between the institution and the students, as well as the students’ integration within the school setting. It is based on variables that effectively contribute to predicting a situation of dropout early enough to address it before it happens.

According to Tinto, the key to the effective retention of students—especially those who perceive a gap between their own culture and the school culture—is found in the strong commitment of institutions to provide quality education and build a cohesive and inclusive environment.

This occurs within the classroom: the educational encounters that take place there are a major feature of students’ educational experiences. If classrooms become learning communities, it is possible to improve students’ involvement, learning, and perseverance. Special attention must thus be devoted to the quality of all classroom relationships.

In this perspective, the suggestion by a group of Danish scholars to consider school dropout as a result of a crisis of trust is therefore especially relevant.

Overall, general trust is described as the positive expectation within a relationship regarding the actions and intentions of others, who are assumed to be reliable [23,24,25]. Research has shown that in societies with high levels of trust, people tend to enjoy better health [26,27]. School trust is defined as the “connective tissue” that unites individuals to promote both education and well-being of students [28], and it manifests at multiple levels: interpersonal relationships between individuals and groups (interpersonal trust), within classrooms (educational trust), and within the broader educational organization (institutional trust, or “school trust”) [29]. School trust translates into the expectations that an individual holds regarding the educational system and how it will contribute to their future development [30].

The recent literature has shown that, since general trust is associated with social well-being [31], students who trust their schools and their teachers show higher levels of social well-being: they tend to exhibit greater self-efficacy [32], higher life satisfaction [33], lower levels of depression [34], and are more likely to engage actively in the learning process, as they feel more supported by their educational environment [35].

General trust is considered an important component of the school climate [36], although its specific features are relatively underexplored.

School climate is a construct grounded in a strongly systemic theoretical framework [37], offering a global and multidimensional perspective on the school environment and aspects related to daily practices, as well as on the relational and educational environment [38]. It is commonly defined as the quality and character of the school [39], and a vast body of literature highlights its associations with better academic outcomes for students and its effect on the mental health of teachers and students alike [40,41].

Specifically, among the dimensions of school climate are the sense of belonging to the school and teachers’ positive teaching. These two aspects seem to be directly connected to trust in school and in teachers.

The sense of belonging is defined as “the need to be and perception of being involved with others at differing interpersonal levels (…) which contributes to one’s sense of connectedness (being part of, feeling accepted, and fitting in), and esteem (being cared about, valued and respected by others), while providing reciprocal acceptance, caring and valuing to others” [42]. This multidimensional construct is a protective factor for social well-being, mental health, and academic outcomes, especially during school transitions [43]. Students who experience a strong sense of belonging to their school tend to have higher levels of engagement, fewer internalizing and externalizing symptoms, and better academic outcomes [44]. Positive teaching refers to teachers’ sense of job satisfaction and the practices they adopt to engage students in their educational experience [45]. It is closely related to the construct of “passion” in education, which is characteristic of teachers who love their subject and their students and are convinced that teaching can make a difference in their students’ lives [46].

Finally, self-efficacy refers to one’s confidence in their ability to successfully perform the actions necessary to achieve a specific goal [47]. In the educational context, self-efficacy describes students’ belief in their ability to achieve specific educational outcomes. In addition to contributing to academic success and reducing the likelihood of school disengagement, it constitutes a psychological resource that supports coping processes in response to academic challenges [48], and it mitigates the impact of gender, race, and ethnicity on the intention to drop out of school [49].

Research suggests that the sources of individual self-efficacy in school can vary (e.g., due to experiences of mastery, emotional states, social contagion between peers, and vicarious experiences), and that self-efficacy influences a range of factors, such as academic performance, self-regulated learning, and perseverance. Perseverance is particularly important for retaining students who consider dropping out of school [48,50].

Self-efficacy is also conceptualized as a psychosocial precursor to emotional engagement, with the sense of belonging being one of its key dimensions [51].

Moreover, studies show that trust, supported by positive emotional warmth from caregivers, enhances adolescents’ self-esteem, which in turn strengthens their self-efficacy [32].

Based on these insights from the literature, the general purpose of this study was to explore the interaction of various relational constructs related to school life, which could be used to improve student well-being and reduce the risk of dropout, in alignment with Tinto’s model. In particular, the study aimed to investigate the role that school trust plays in relation to students’ sense of belonging to the school, clarifying its interactions with academic self-efficacy and positive teaching.

Indeed, the literature has explored the relationship between interpersonal trust and coping self-efficacy, while the link between students’ trust in teachers and academic self-efficacy has yet to be investigated. Furthermore, the connection between the sense of belonging and school trust has not been thoroughly explored. We hypothesized that the sense of belonging is predicated on a foundation of trust with teachers, which serves as a prerequisite for feeling seen, accepted, respected, cared for, and valued by the key adults within the educational environment [42].

Based on the above, the following research hypotheses were developed:

**H1:**  *Students’ trust in teachers has a positive correlation with academic self-efficacy, positive teaching, and the sense of belonging to the school.*

**H2:**  *Positive teaching has a positive correlation with academic self-efficacy, students’ trust in teachers, and the sense of belonging to the school.*

**H3:**  *Students’ trust in teachers, academic self-efficacy, and positive teaching positively predict the sense of belonging.*

**H4:**  *The relationship between positive teaching and the sense of belonging is sequentially mediated by trust in teachers and academic self-efficacy.*

The results suggest that a school climate built on trust between students and teachers can reduce the dropout risk through its impact on the sense of belonging, a component of school climate and emotional engagement. Thus, interventions should focus on enhancing trust relationships and teachers’ relational skills, especially in disadvantaged contexts. Indeed, school trust represents a crucial factor that surpasses social and cultural differences.

## 2. Materials and Methods

### 2.1. Participants and Procedure

The study involved five secondary schools from the eastern suburbs of Naples, in the Campania region. These schools, which at the time were involved in various projects against school dropout, accepted our invitation to participate in the research. In this region, located in Southern Italy, the percentage of early school leavers is about 16% (national average = 10.5%), while the average number of NEETs is around 26.9% (national average = 16.1%) [52]. In particular, the eastern area of the City of Naples is characterized by high population density, one of the highest in Europe, along with a range of social issues. These include housing shortage, high unemployment and informal labor, family distress, educational poverty, and juvenile delinquency.

The study focused on 41 9th- and 10th-year classes, since these years represent a potentially critical period for students [17,53].

Participation in the study was anonymous and voluntary. Informed consent was obtained from the parents of the students, as they were minors. The informed consent form provided detailed information on the study’s aims, procedures, and the confidentiality and anonymity of the data. The Research Ethics Committee (CERP) of the Department of Humanities (DSU) at the University of Naples “Federico II” approved the study. Teachers, educators, and researchers from the DSU supervised the administration of the questionnaires during school hours, providing students with relevant information and supporting them with any difficulties encountered.

Data were collected through an online survey administered via the Qualtrics Experience Management (XM) platform, which assessed the variables of interest.

A total of 645 participants (M = 324, F = 321) aged 13 to 17 years (M_age_ = 14.6, SD = 0.69) completed the questionnaire. Since questionnaires were administered during school hours, the students who had dropped out were not included in this administration. However, they were involved in a qualitative study that is part of the broader research and that is currently in the process of publication.

### 2.2. Instruments and Measures

**Self-Efficacy–Academic Perceived Self-Efficacy Scale (APSES).** The APSES [54] is a self-report scale consisting of 15 items that assess students’ beliefs in their ability to master different school subjects, regulate their motivation and learning activities, and meet the expectations of parents and teachers. In particular, the participants are asked how confident they feel in a range of areas described by the items. Examples of the items include “Finishing homework assignments on time”, “Meeting the expectations of your teachers”, and “Meeting the expectations of your parents regarding what they expect from you”. This scale shows good internal consistency, with Cronbach’s α around 0.80. In the present study, Cronbach’s α was 0.88.

**Trust in Teachers–Student Trust in Teachers Scale (STT).** The students’ trust in teachers was measured using the scale developed by Adams and Forsyth [55], which consists of 13 items. The scale is divided into 5 subscales: benevolence, competence, reliability, honesty, and openness. Examples of the items include “It is easy to talk to the teachers in this school”, “The teachers of this school have high expectations for all students”. This scale shows good internal consistency, with Cronbach’s α around 0.90. In the present study, Cronbach’s α was 0.88.

**Positive Teaching–Multidimensional School Climate Questionnaire (MSCQ).** Positive teaching was assessed using the scale from the MSCQ in its Italian version [45]. The scale consists of 5 items. Examples of the items are “Most of the teachers seem to teach with pleasure”, “Most of the teachers seem to really love their job”, “The teachers use methods that make the subject interesting”. The Italian version of this scale shows good internal consistency, with Cronbach’s α = 0.78. In the present study, Cronbach’s α was 0.83.

**Sense of Belonging–MSCQ.** To measure the sense of belonging, we also used the corresponding MSCQ scale [45], which includes 5 items. Examples of the items are “I would prefer to be in another school”, “I love my school”, “This school is important to me”. The Italian version of this scale shows good internal consistency, with Cronbach’s α = 0.88. In the present study, Cronbach’s α was 0.85.

**Sociodemographic variables.** The sociodemographic control variables were the parents’ educational level (1 = elementary school, 2 = middle school, 3 = high school diploma, 4 = university degree) and the students’ academic failures (1 = no, 2 = yes). This information was collected via the self-report questionnaire.

### 2.3. Statistical Analyses

Various statistical analyses were conducted in order to analyze the collected data and test the hypotheses of the present study. These analyses were performed using the SPSS 29.0.1.0 package (IBM Corp, Armonk, NY, USA) and the PROCESS macro extension by Hayes.

In particular, correlation analyses were conducted to examine the relationships between students’ trust in teachers (STT), academic self-efficacy (EFSC), positive teaching (PT), and the sense of belonging (SB). Moreover, hierarchical multiple regression analyses were carried out to assess the predictive effect of each independent variable on the sense of belonging (SB). Finally, mediation analyses were performed to evaluate the mediating roles of students’ trust in teachers (STT) and academic self-efficacy (EFSC) in the relationship between positive teaching (PT) and the sense of belonging (SB).

## 3. Results

### 3.1. Descriptive Statistics and Group Differences

The means and standard deviations of the variables examined, as well as the results of the t-test analyzing gender differences and Cronbach’s α coefficient, are reported in Table 1.

The means for positive teaching (PT) and the sense of belonging (SB) were 17.8 (SD = 4) and 12.9 (SD = 2.4), respectively; for trust in teachers (STT), the mean was 32.7 (SD = 5.7), and for academic self-efficacy (EFSC), it was 47.5 (SD = 9.7).

As shown in Table 1, the t-test highlighted differences between the students who had not experienced grade retention and those who had been held back at least once in academic self-efficacy (EFSC). Specifically, the non-retained students reported higher scores compared to the retained students (t_(633)_ = 3.938, *p* < 0.001). However, no significant differences were found for the other variables under investigation, and gender differences had no significance at all.

The ANOVA revealed significant differences among the groups based on the parents’ educational level (PEL). Firstly, significant differences were found in the academic self-efficacy (EFSC) scores (F_(6, 606)_ = 4.813, *p* < 0.001, η^2^ = 0.04), with a small effect size. Levene’s test confirmed the homogeneity of variances for EFSC (*p* = 0.407), ensuring the validity of the ANOVA results. Post hoc comparisons using Tukey’s HSD test indicated that significant differences in the EFSC scores were observed between the students whose parents had a middle school diploma and those whose parents held a university degree, with the children of more highly educated parents reporting significantly higher self-efficacy (*p* < 0.001). This suggests that higher parental education, particularly at the university level, is associated with greater academic self-efficacy in students.

Additionally, the positive teaching (PT) scores also varied significantly across the PEL groups (F_(6, 532)_ = 2.481, *p* < 0.05, η^2^ = 0.02). The test of homogeneity indicated no violation of variance assumptions (*p* = 0.605). Post hoc analysis revealed significant differences between the students whose parents had an elementary school diploma and those whose parents had a university degree, with the students whose parents had only an elementary school education level reporting a lower perception of positive teaching practices (*p* = 0.042). This implies that parental education influences students’ perceptions of their teachers, with higher levels of parental education being associated with more positive views of teaching practices.

Conversely, no significant differences were observed in the trust in teachers (STT) scores (F_(6, 478)_ = 1.617, *p* = 0.14, η^2^ = 0.01), and the variances were homogeneous (*p* = 0.737), as indicated by Levene’s test. Therefore, parental education does not seem to have a significant impact on students’ trust in their teachers.

Similarly, no significant differences emerged regarding the students’ sense of belonging to the school (SB) (F_(6, 523)_ = 1.155, *p* = 0.33, η^2^ = 0.01). However, Levene’s test revealed a marginal violation of the assumption of homogeneity for the SB (*p* = 0.040). This suggests that caution should be exercised when interpreting the ANOVA results for the SB, as the lack of homogeneity of variances might have affected the test’s robustness.

### 3.2. Correlations

The correlations among the study variables are presented in Table 2. The results revealed that:

- Parents’ educational level (PEL) shows a significant negative correlation with academic failures (*r* = −0.1) and a significant positive correlation with academic self-efficacy (*r* = 0.19);
- Academic failure has a significant negative correlation with academic self-efficacy (*r* = 0.14);
- Academic self-efficacy has a significant positive correlation with positive teaching (*r* = 0.22), students’ trust in teachers (*r* = 0.27), and the sense of belonging (*r* = 0.32);
- Positive teaching shows a significant positive correlation with students’ trust in teachers (*r* = 0.65) and the sense of belonging (*r* = 0.48).
- The sense of belonging has a significant positive correlation with students’ trust in teachers (*r* = 0.56).

### 3.3. Hierarchical Multiple Regression Analyses

Hierarchical multiple regression analyses were conducted to determine the extent to which some of the analyzed variables contribute to the explanation of the sense of belonging to the school (SB). The aim of this study was to explore the contribution of each variable, and especially to evaluate the effect of the relational variables within the school; consequently, this type of analysis was chosen in order to insert, in a hierarchical sequence, first the sociodemographic control variables (PEL and AF), then the individual variables, and lastly the relational ones.

The resulting model is described in Table 3.

After controlling for the effects of parents’ education level (PEL) and grade retention, the variables academic self-efficacy (EFSC), positive teaching (PT), and students’ trust in teachers (STT) were introduced sequentially. The inclusion of EFSC resulted in an R^2^ increase of 0.102. Adding PT led to an additional R^2^ increase of 0.195, and finally, the inclusion of STT resulted in an R^2^ increase of 0.074. The complete model, which includes parents’ education level, grade retention, EFSC, PT, and STT, was statistically significant, with an R^2^ of 0.614.

### 3.4. Mediation Analyses

**Figure 1:**
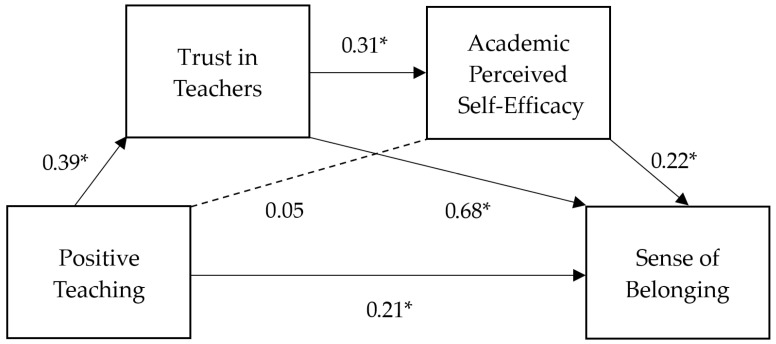
Mediation model with coefficients for direct effects. *N* = 645. Note: * *p* < 0.001. All the present effects are unstandardized.

The mediation analysis, conducted using Model 6 of the PROCESS macro, aimed to investigate the multiple mediating roles of trust in teachers and academic self-efficacy in the relationship between positive teaching and the sense of belonging to the school. The results revealed significant relationships between the variables examined. Figure 1 illustrates the mediation model and shows the coefficients for the observed direct effects.

Firstly, the direct effect of positive teaching (PT) on the sense of belonging (SB) was significant (β = 0.21, *p* < 0.001), but smaller than the total model effect, suggesting that this effect may be partially mediated by the other variables considered. Specifically, the indirect effect of positive teaching (PT) on the sense of belonging (SB) through the mediation of trust in teachers (STT) was significant (β = 0.2600, BootLLCI = 0.1922, BootULCI = 0.3282). However, the pathway PT → EFSC → SB was not significant (β = 0.0140, BootLLCI = −0.0083, BootULCI = 0.0410), indicating that academic self-efficacy (EFSC) alone does not mediate the relationship between positive teaching (PT) and the sense of belonging (SB).

On the other hand, the final model PT → STT → EFSC → SB was significant (β = 0.0279, BootLLCI = 0.0105, BootULCI = 0.0497), supporting the sequential mediation effect where positive teaching (PT) influences the sense of belonging (SB) through trust in teachers (STT), which subsequently enhances the effect of academic self-efficacy (EFSC) and ultimately increases the sense of belonging (SB). Among the indirect pathways described, the most significant role is played by trust in teachers (STT), which mediates a substantial portion of the effect of positive teaching (PT) on the sense of belonging (SB).

Finally, the overall mediation effect of positive teaching (PT) on the sense of belonging (SB) through trust in teachers (STT) and academic self-efficacy (EFSC) was significant (β = 0.3019, BootLLCI = 0.2341, BootULCI = 0.3696) and accounted for a considerable portion of the total effect of PT on the SB (approximately 57%).

In summary, the findings indicate that positive teaching (PT) influences the sense of belonging (SB) not only directly, but predominantly through students’ trust in their teachers. Additionally, academic self-efficacy (EFSC) plays an important role in this relationship when considered in a sequential model with trust in teachers (STT), but it does not show significance when examined independently.

Details on the mediation model are given in Table 4.

## 4. Discussion

The scientific literature suggests that school dropout is the result of a deterioration in the bond between individuals, schools, and society [2,3]. This may be due to the accumulation of push factors, characteristics inherent in the school system, or the relationship between the school and the students, which all contribute to pushing the latter to drop out of their educational path; and pull factors, i.e., factors external to the school—such as parental expectations, family problems, trauma, and relationships with “undesirable peers”—which can “pull” students out of the educational system [56]. While pull factors seem to be more relevant than push factors in predicting dropout [57], the latter still play a crucial role and, most importantly, help clarify the internal aspects of the educational system that could be addressed through reforms or targeted programs, as highlighted in Tinto’s model.

School trust is described as the connective tissue that binds relationships within the school. Several studies emphasize its role in promoting behavioral engagement and self-esteem; however, there is limited evidence regarding its connection to the affective components of engagement, such as the students’ sense of belonging to the school. The sense of belonging, a component of the school climate, is itself a protective factor against the risk of dropout [44], and is positively correlated with higher levels of self-efficacy.

Research on school dropout tends to focus on individual factors, resulting in an extensive body of data on student-related variables. The present study aimed to investigate relational dimensions associated with dropout, particularly focusing on students’ trust in teachers. The primary goal of the study was to explore the relationships between students’ trust in teachers and two components of the school climate (teachers’ positive teaching and the students’ sense of belonging), observing their interaction with a student-related factor, which has emerged as relevant in the literature on school dropout and school trust (academic self-efficacy). Specifically, the study sought to examine the role that trust in teachers plays in fostering the students’ sense of belonging to the school, especially in interaction with the other variables under investigation.

The findings of the study confirmed all the hypotheses, although H4 was only partially supported.

The results of the correlation analyses supported both H1 and H2, showing that trust and positive teaching were positively correlated with each other, as well as with academic self-efficacy and the sense of belonging. Positive teaching was significantly associated with both trust in teachers and the sense of belonging.

These data show how a cycle of salutogenesis can develop within a positive educational environment when a combination of positive dimensions occurs, such as, on the one hand, adults who appear trustworthy and teach with enthusiasm, and, on the other hand, adolescents who feel capable of growth and feel a sense of belonging to their school.

According to some studies, indeed, a teacher should be able to perform both a maternal function, providing reliability and psychological security, and a paternal function, acting as a model of an adult who has passion and enthusiasm for their work. Only in this way can an adolescent feel that the school is their home and view learning as a challenging yet promising endeavor with a high likelihood of success [58].

Moreover, H3 was confirmed by the hierarchical multiple regression analyses. Although the study lacks longitudinal data, trust in teachers, academic self-efficacy, and positive teaching emerged as potential predictors of the students’ sense of belonging to the school. These findings appear to add a new perspective on the relationship between self-efficacy and the sense of belonging, as it was the students’ self-efficacy that predicted the sense of belonging to the school. It can be inferred that feeling capable of learning and achieving good results contributes to feeling like active participants, not marginal figures, in the life of their school.

Finally, H4 was partially supported. Mediation analyses suggested that the direct effect of positive teaching on the sense of belonging was amplified by trust in teachers. However, academic self-efficacy alone was not a significant mediator in the relationship between positive teaching and the sense of belonging. Instead, self-efficacy became a significant mediator when considered in sequential interaction with trust in teachers. Therefore, trust in teachers appears to mediate not only the relationship between positive teaching and the sense of belonging, but also the relationship between positive teaching, academic self-efficacy, and the sense of belonging. This highlights the centrality of trust in fostering the sense of belonging: how could students feel that the school is their home if there are adults within it whom they cannot trust? This finding is of particular interest because it has significant practical implications: it is possible to work with teachers by providing them with both educational and psychological support to improve their well-being, a fundamental prerequisite for their ability to “earn” students’ trust. Especially in certain complex, marginalized contexts, teachers often report feelings of isolation and ineffectiveness, sometimes even leading to burnout. It is essential to provide them with tools that foster cooperation and reflection to help them cope with their high-risk work, which is prone to psychological distress [59,60].

Regarding group differences in the control sociodemographic variables, grade retention was associated with lower levels of academic self-efficacy, consistent with the literature, while it was not significant for the other variables examined. The lack of relevance of the sex of the participating students is of considerable interest, considering its usual weight in studies on school dropout and adolescent mental health [20]. Concerning the parents’ education level, t-tests revealed that this factor was relevant for explaining differences in scores for academic self-efficacy and positive teaching, but it was not significant for trust in teachers or the sense of belonging. It seems that trust belongs to a “supraordinate” dimension, which relates more to the characteristics and attitudes of adults than to cultural differences or specific conditions of adolescents.

In conclusion, the findings suggest that schools should focus on fostering trust-based relationships between students and teachers. This includes supporting teachers in developing their skills and enhancing their ability to share information transparently (openness), demonstrate clarity and consistency (reliability), acknowledge their mistakes (honesty), and show understanding of their students’ interests (benevolence) [24,61,62,63,64]. Furthermore, it is essential to promote the well-being of teachers: although the literature shows that “happy teachers make happy students” [65], teachers’ well-being is still not considered a priority area for research, let alone intervention [66]. Our data, however, suggest that investing in policies that promote the organizational and relational well-being of teachers, in addition to their continuous professional development, could be a strategic and even economical choice to prevent school dropout. After all, every teacher has the ability to inspire trust in an entire class. This is particularly important in marginalized contexts with high dropout rates, such as the one in our study, where teachers, in turn, need to trust the educational institution and receive adequate support to face the challenges related to the area’s educational poverty.

The United Nations 2030 Agenda also placed among its Sustainable Development Goals a target to substantially increase the supply of qualified teachers by 2030 [67]. “Qualified teachers” means, concretely, teachers who are capable of working on themselves through the methods that psychological research has long indicated as effective, such as reflective groups conducted by expert psychologists [68], as well as teachers who are capable of working in groups with other professionals in the community, becoming part of support networks and building positive alliances [14]. Unfortunately, in Italy, the lack of a national law on school psychologists has fostered emergency and restorative interventions mainly aimed at dropout students. However, the data collected by this study show that the right road to follow is one of stable cooperation between education professionals, aimed at creating school communities that are cohesive, but also open and flexible, and where priority is given to the care of bonds and everybody’s well-being. “*Only through such diverse, long-term, and sustained effort and intervention, and not through any single, concentrated all-out effort or dramatic, heroic remedy, will it be possible to create a school and a learning environment dominated by factors of presence, eliminating more of the factors of absence, and thereby increasing completion in upper secondary school, and reducing the group that ends up in danger of entering a marginalized position in relation to employment and social life*” [14] (p. 228).

### Strenghts, Limitations, and Future Directions

To our knowledge this study is the first to explore the link between students’ trust in teachers, academic self-efficacy, positive teaching, and the sense of belonging to the school.

In particular, the decision to focus on students from the disadvantaged outskirts of Naples adds significant value to this research, since marginalized communities are rarely involved in school dropout studies [1]. With respect to the existing literature, the results of this study thus contribute to broadening knowledge on the phenomenon of school dropout, highlighting the crucial contribution of relational factors within the school system. These could counter the defensive mechanism of exit from a context that is felt as unwelcoming regardless of the sociodemographic characteristics of the individual [14]. Thus, the current study provides significant insights into the challenges that students have to face in these contexts, informing targeted interventions in similar socioeconomic environments.

Moreover, positive teaching represents a dimension of school climate [45] that has been underexplored in the literature, even though it offers valuable insights into how teachers approach their profession and interact with their students. This becomes particularly relevant when considering the tendency of research and interventions to adopt a reparative perspective, often focusing on students without considering the broader context and relationships in which they are situated [1,69,70].

Nonetheless, the cross-sectional design of this study prevents researchers from establishing causal inferences. Additionally, the geographically limited sample reduces the generalizability of the results. Moreover, the choice of schools involved in the projects aimed at countering school dropout might have created some form of bias; nevertheless, as these projects did not involve the teachers directly and were held during extracurricular hours, it is possible that, at most, in some cases they might have helped students to reflect on the similarities and differences between school and out-of-school experiences.

Finally, as it is quantitative in nature, the study does not delve into the psychological and social dynamics and experiences influencing students. Based on the above, future research could gain a more comprehensive understanding of the factors driving school engagement and dropout risks by adopting a qualitative approach.

Moreover, future inquiries could observe intervention-based studies to understand how fostering student–teacher trust or improving academic self-efficacy through specific programs can reduce dropout rates. It would also be important to focus on the relationships with peers and classmates, given the key role that they play in this life phase. Finally, researchers could also consider teachers’ perspectives on the student–teacher relationship and how they experience their profession. Understanding how teachers perceive their own roles could inform professional development programs aimed at improving their relational skills.

## 5. Conclusions

The important social and economic changes that characterize our society have caused a crisis of psychological and social meta-guarantors [71,72], including the school institution [73,74]. In this scenario, the representation that students build of their school and their teachers is especially relevant [75].

Thus, the results of this study, which have shown the significant role of students’ trust in teachers in relation to their sense of belonging to the school, open the door to further and important reflections. In particular, they confirm that interventions against school dropout should adopt both programmatic and systemic strategies [70]: while programmatic interventions attempt to influence students’ behaviors, thoughts, values, and feelings, the systemic ones should enhance the educational and relational environment.

If a young person has the misfortune of being born into a context that offers limited resources for growth, they have a specific need to encounter significant adults capable of fostering trust in others and in a better future. Nonetheless, the competence, benevolence, openness, and honesty of adults and teachers must be fostered by the institution itself, which should provide adequate training for professional development.

## Figures and Tables

**Table 1:** Descriptive analyses and group differences.

	Total Sample (*N* = 645)		M (*N* = 324)		F (*N* = 321)			Non-Academic Failures (*N* = 590)		Past Academic Failures (*N* = 55)			
	M	SD	M	SD	M	SD	t	M	SD	M	SD	*t*	*α*
EFSC	47.5	9.7	3.1	0.6	3.2	0.7	−1.5	3.2	0.6	2.9	0.5	3.9 **	0.88
PT	17.8	4	3.5	0.8	3.5	0.8	−1.1	3.5	0.8	3.6	0.8	−0.1	0.85
STT	32.7	5.7	2.7	0.4	2.7	0.5	0.8	2.7	0.4	2.6	0.5	1.1	0.88
SB	18	4.2	3.6	0.8	3.5	0.8	0.6	3.6	0.8	3.5	0.7	0.5	0.83

EFSC = academic self-efficacy; PT = positive teaching; STT = students’ trust in teachers; SB = sense of belonging. Note: ** *p* < 0.01.

**Table 2:** Correlation analyses.

	1	2	3	4	5	6
PEL	-					
2. AF	−0.1 *	-				
3. EFSC	0.19 **	−0.14 **	-			
4. PT	−0.10 *	0.01	0.22 **	-		
5. STT	0.03	−0.05	0.27 **	0.65 **	-	
6. SB	−0.04	−0.02	0.32 **	0.48 **	0.56 **	-

PEL = parents’ educational level; AF = academic failure; EFSC = academic self-efficacy; PT = positive teaching; STT = students’ trust in teachers; SB = sense of belonging. Note: * *p* < 0.05, ** *p* < 0.01.

**Table 3:** Hierarchical multiple regression analysis for the prediction of the sense of belonging (*N* = 645).

Predictors	*B*	SE	β	t	*p*	R^2^	∆R^2^
Step 1						0.005	
PEL	0.048	0.058	0.038	0.835	0.404		
AF	−0.187	0.147	−0.590	−1.273	0.204		
Step 2						0.108	0.102
EFSC	0.430	0.058	0.328	7.377	<0.001		
Step 3						0.302	0.195
PT	0.479	0.042	0.459	11.521	<0.001		
Step 4						0.377	0.074
STT	0.643	0.085	0.369	7.524	<0.001		

PEL = parents’ educational level; AF = academic failure; EFSC = academic self-efficacy; PT = positive teaching; STT = students’ trust in teachers.

**Table 4:** Mediation analysis of positive teaching on the sense of belonging through students’ trust in teachers and academic self-efficacy (*N* = 645).

Path	Effect	BootLLCI	BootULCI	SE	*T*	*p*-Value
**Total effect**	0.5194	0.4383	0.6005	0.0413	12.5836	<0.001
**Direct effects**						
PT → SB	0.2175	0.1197	0.3153	0.0498	4.3692	<0.001
PT → STT	0.3923	0.3520	0.4326	0.0205	19.1386	<0.001
PT → EFSC	0.0592	−0.0304	0.1487	0.0456	1.2988	0.195
STT → EFSC	0.3008	0.1518	0.4499	0.0758	3.9662	<0.001
STT → SB	0.6627	0.1197	0.3153	0.0498	4.3692	<0.001
EFSC → SB	0.2365	0.1399	0.3331	0.0492	4.8118	<0.001
**Indirect effects**						
PT → STT → SB	0.2600	0.0357	0.1935	0.3318		
PT → EFSC → SB	0.0140	0.0120	−0.0080	0.0388		
PT → STT → EFSC → SB	0.0279	0.0098	0.0500	0.0100		

EFSC = academic self-efficacy; PT = positive teaching; STT = students’ trust in teachers; SB = sense of belonging.

## Data Availability

The data presented in this study are available upon request from the corresponding author.
